# Regulation of osteogenic differentiation in vascular smooth muscle cells under high-glucose condition

**DOI:** 10.3389/fendo.2025.1589160

**Published:** 2025-09-05

**Authors:** Jingjing Guo, Laijing Du

**Affiliations:** ^1^ Luoyang Key Laboratory of Cardiovascular Science, the First Affiliated Hospital, and College of Clinical Medicine of Henan University of Science and Technology, Luoyang, China; ^2^ Department of Cardiology, Henan Key Laboratory of Cardiovascular Science, the First Affiliated Hospital, and College of Clinical Medicine of Henan University of Science and Technology, Luoyang, China

**Keywords:** atherosclerosis, calcification, osteogenic transformation, vascular smooth muscle cells, hyperglycemia research insights

## Abstract

Diabetic patients have a higher tendency for vascular calcification (VC). This indicates a possible link between abnormal glucose metabolism and the development of VC. High glucose levels are a major cause of vascular calcification in diabetic patients. Vascular smooth muscle cells (VSMCs) are important functional units of the arterial media and show heterogeneity. Sustained hyperglycemia drives VSMCs to undergo a phenotypic transition from contractile state to osteo-/chondrogenic lineages through multiple pathophysiological mechanisms. Specifically, hyperglycemia stimulates metabolic reprogramming. This includes enhancing advanced glycation end products (AGEs), activating the diacylglycerol-dependent protein kinase C (PKC) pathway, disrupting the pentose phosphate flux (PPP), and dysregulating the hexosamine biosynthesis pathway (HBP). These changes trigger vesicles-mediated mineralization (including matrix/extracellular vesicles), oxidative stress, inflammatory cascades, and an imbalance between autophagy and apoptosis. This review systematically describes the metabolic remodelling induced by high glucose and its regulatory mechanisms in vascular calcification.

## Highlights

What is currently known about this topic?

- Diabetic patients face a higher risk of coronary artery calcification, which is related to hyperglycemia.
- VSMCs play a key role in vascular calcification and can differentiate into osteoblast-like cells.
- VSMCs osteogenic differentiation is likely driven by specific diabetes mellitus–associated mechanisms. These include oxidative stress, PKC activation, PPP, and HBP. These processes regulated by key enzymes such as hexokinase 2 (HK2), pyruvate kinase M (PKM), β-N-acetylglucosaminidase (OGA) and β-N-acetylglucosaminyltransferase (OGT).

What is the key research question?

- How does hyperglycemia induce the osteogenic differentiation of VSMCs?
- what are the underlying molecular mechanisms?

What is new?

- Elucidation of the complex regulatory network of metabolic reprogramming in VSMCs under diabetic conditions.
- Discovery of the role of various microRNAs and extracellular vesicles in regulating VSMCs osteogenic differentiation.

## 1 Introduction

Forecasts indicate that by 2030, 552 million people will be diabetes (1–3) Cardiovascular calcification was found in 81.2% of diabetic patients and 33.7% of nondiabetic patients. Therefore, diabetic patients are highly prone to VC (4, 5). A better understanding of the molecular processes between high glucose and VC may accelerate the development of new biomarkers and targeted drugs for calcification.

VC is mainly divided into initial and medial calcification (6). VSMCs are important components of the vascular media. In diabetes, hyperglycemia disrupts redox homeostasis and triggers inflammatory signaling, oxidative stress, formation of Ca-Pi crystals, O-GlcnAcylation, promoting the osteogenic differentiation of VSMCs (7–10).

During the osteogenic differentiation of VSMC, hyperglycemia upregulates expression of osteopontin (OPN) and osteoprotegerin (OPG) and then activates Msh homeobox-2 (MSX2) and Runt-related transcription factor 2 (RUNX2) through the wingless - type MMTV integration site family (WNT)/β-catenin and bone morphogenetic protein-2 (BMP-2) pathways (11–14). The mechanisms of VSMCs calcification caused by diabetes have been a hot topic of research in recent years, which mainly include oxidative stress, inflammation, death regulation, matrix vesicle formation, mineral deposition (15–17).Under the high glucose condition, glucose metabolic reprogramming is crucial for the phenotypic transformation of VSMCs and contributes to vascular remodeling (18–20). The complex interaction between metabolic reprogramming, signaling pathway activation, cell fate decisions, and post-transcriptional regulation underlies the osteogenic differentiation of VSMCs in diabetes. Besides, high glucose impairs the pyrophosphate-to-phosphate ratio and induce calcium deposition, disrupts extracellular pyrophosphate and calcium metabolism (21). Despite some progress in recent years, the impact of hyperglycemia on VSMCs osteogenic differentiation remains unclear. In-depth studies are needed to clarify the detailed mechanisms of VSMCs osteogenic differentiation in diabetes. Therefore, this reviewer pays attention to the pathophysiology of VSMCs osteogenic differentiation induced by hyperglycemia.

## 2 VSMCs’ osteogenic differentiation: a key event in VC

**Figure 1:**
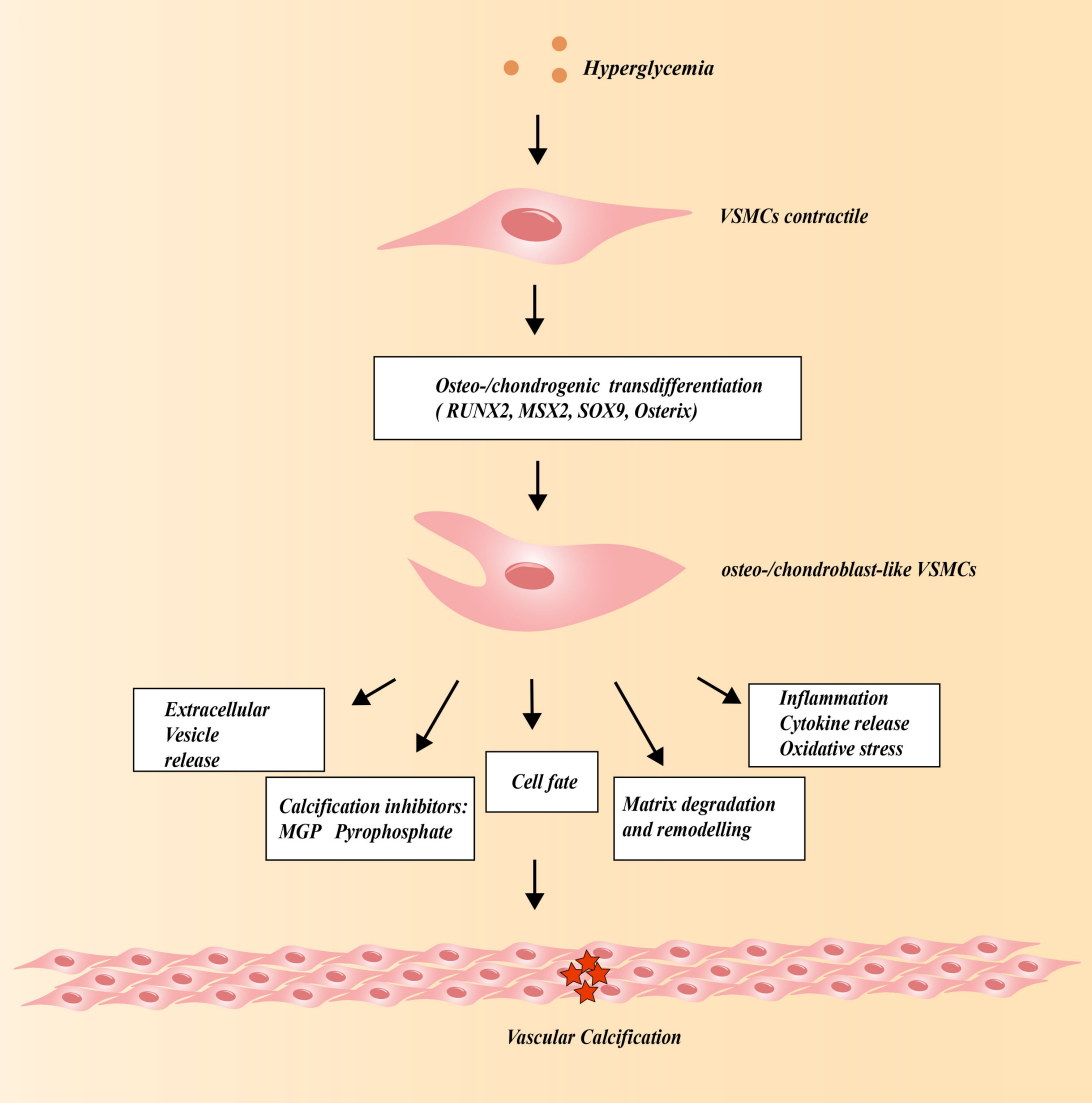
The role of VSMCs in Vascular calcification under the high glucose condition.

The etiology of VC involves the osteogenic differentiation of VSMCs (22). VSMCs exhibit phenotypic plasticity (23). With the development of high-throughput detection technologies such as single-cell and transcriptomics sequencing analysis, it is realized that VSMCs can be transformed into pro-inflammatory chemotactic like, macrophage-like/foam-like, and fibroblast/chondroblast-like smooth muscle cells (24–26). Among them, the osteogenic transformation of VSMCs is an important link in vascular calcification. During VSMCs’ osteogenic differentiation, the levels of MSX2, BMP2, RUNX2, osterix, sex-determining region Y-box 9 (SOX9) increase. These upregulate osteocytic and chondrocyte markers [such as OC (osteocalcin), Col1α1 (collagen type I α1), OPN, and alkaline phosphatase (ALP)] and downregulates contractile markers like smooth muscle 22 α (SM22α), calponin, and smooth muscle myosin heavy chain (SM-MHC) (27–31) (**Figure 1**). With the deepening of scientific research, a large number of regulatory factors will be discovered, which will provide new therapeutic targets for osteogenic transformation of VSMCs. For example, high-mobility group box-1 (HMGB-1) promotes vascular calcification in diabetic mice via endoplasmic reticulum stress (15). Non-POU domain-containing octamer-binding protein (NONO) or octamer-binding transcription factor 4 (OCT4) directly bound to the BMP2 promoter and inhibited BMP2 transcription, which protected against osteogenic differentiation of VSMCs (32, 33).

## 3 Glucose metabolic reprogramming and VSMCs osteogenic differentiation

**Figure 2:**
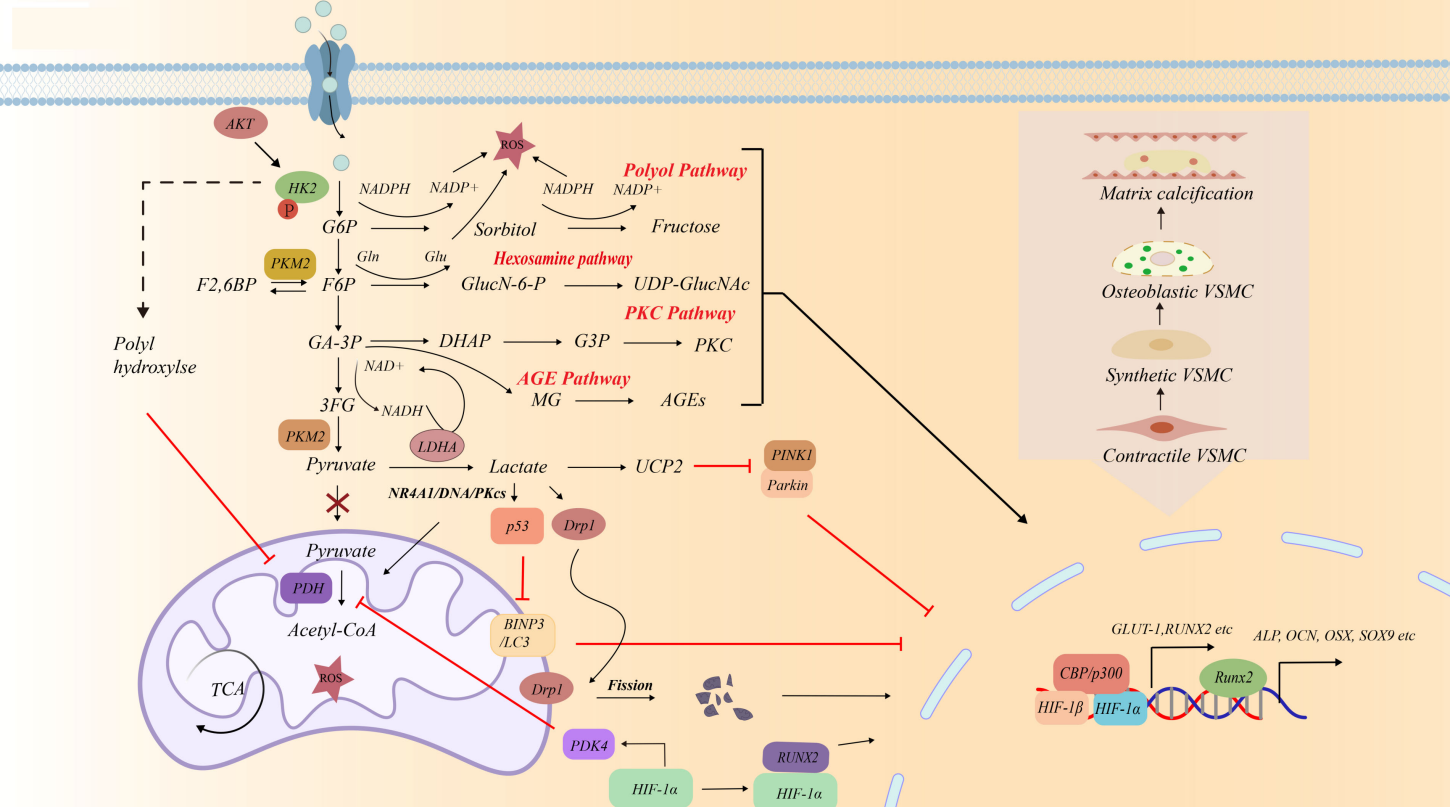
The contribution of unscheduled glycolysis to osteogenic differentiation of vascular smooth muscle cells.

Diabetic patients display elevated glucose in the blood, which lead to VSMCs dysfunction and significantly alter their metabolism. Previous studies have shown that in calcified VSMCs, glucose consumption and lactate generation increase due to a shift towards glycolysis (34, 35). During this process, high glucose promotes glucose uptake through glucose transporter 1 (GLUT1) and reprograms glucose metabolism by regulating activity of HK2, PDK4, 6-phosphofructokinase isozyme 1 (PFK1), PKM2, and lactate dehydrogenase A (LDHA) in VSMCs (12) (**Figure 2**). Therefore, clarifying these metabolic changes could help identify new therapeutic targets for vascular calcification.

### 3.1 GLUT1: the gateway for glucose uptake in VSMCs

**Figure 3:**
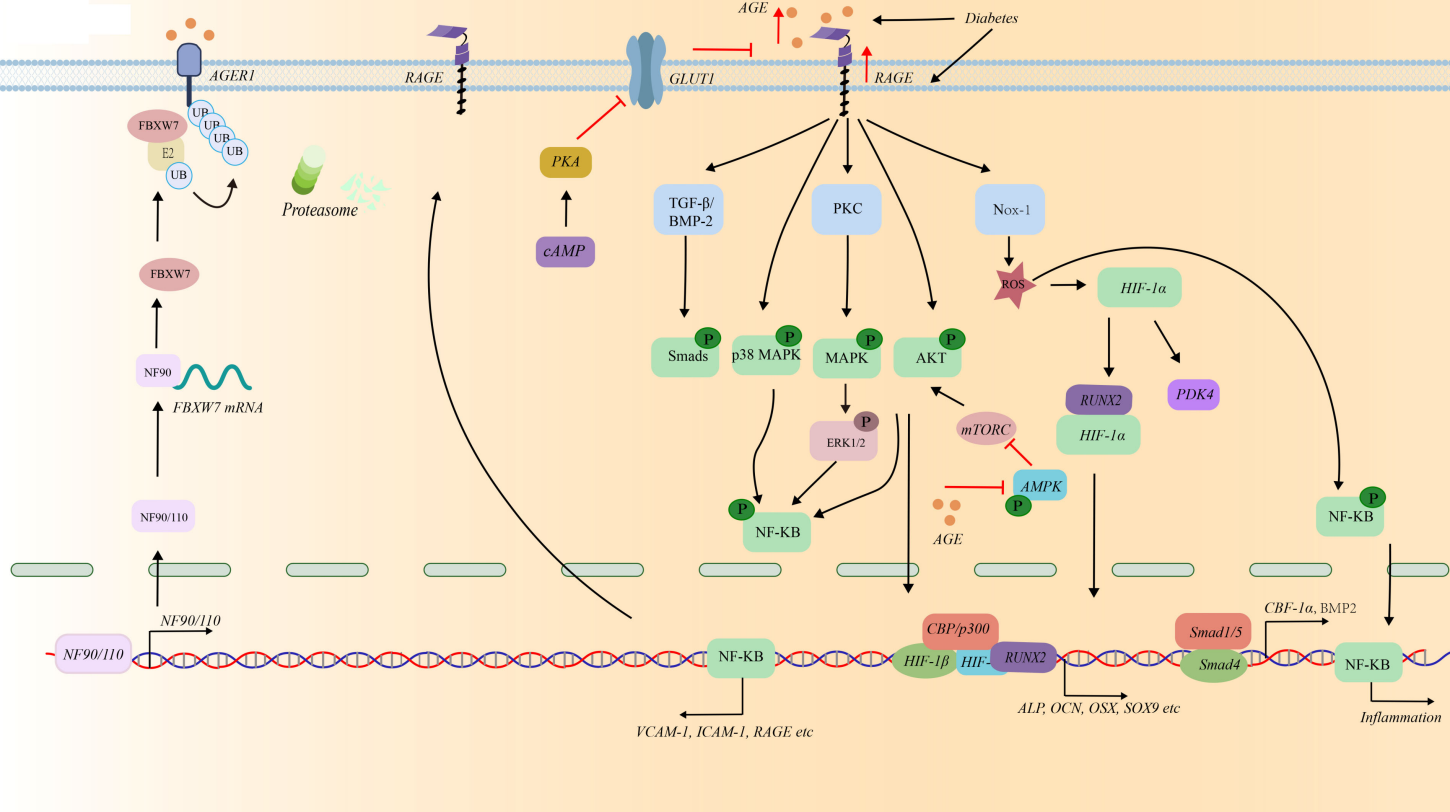
The role of AGEs/RAGE in mediating osteogenic differentiation of VSMCs under the high glucose condition.

GLUT1 is the main isoform of the facilitative GLUT family and mediates glucose uptake in VSMCs (36). It is highly expressed in calcified VSMCs (37). In rodent human arterial smooth muscle cells (HASMCs) and aortic smooth muscle cell lines (A7r5), GLUT1 overexpression increased the intracellular glucose concentration by 44% and enhanced glycolytic flux and tricarboxylic acid cycle activity (TCA) (38–41). Hyperglycemia increase the GLUT1 expression (42). Downregulating GLUT can prevent excessive glucose influx, reducing intracellular protein glycation and free radical generation; both of these are harmful in the development of vascular disease in diabetes (43). High Mobility Group Box 2 (HMGB2) decreased GLUT1 expression and promoted GLUT4 translocation through PPAperoxisome proliferator-activated receptor-γ (PPAR-γ)/silent mating type information regulation 2 homolog 1(SIRT1) (44). Glycogen synthase kinase-3 (GSK-3), an enzyme that hinders the conversion of glucose to glycogen, can inhibit GLUT1 expression and glucose uptake through the tuberous sclerosis complex subunit 2 (TSC2)/mammalian target of rapamycin (mTOR) pathway (38). Ya-Rong Zhang et al. highlighted that intermedin alleviated diabetic vascular calcification by inhibiting GLUT1 through the activation of cyclic AMP (cAMP)/protein kinase A (PKA) signaling pathway (42)(**Figure 3**; **Table 1**). Therefore, GLUT1 serves as the first line of defense against VSMCs calcification induced by high glucose. Therefore, developing targets for inhibiting the regulatory expression of GLUT1 is of great clinical significance.

**Table 1:** Glucose metabolic pathways involved in VSMCs osteogenic differentiation.

Metabolic Pathways	Key Molecules/Enzymes	Biological Effect	Related Research Examples/Mechanisms
Glucose uptake	GLUT1	Increased glucose uptake intracellular protein glycation and free radical generation	1 HMGB2 decreased GLUT1 expression through PPAR-γ/SIRT1 (44). 2 GSK-3 can inhibit GLUT1 expression and glucose uptake throughTSC2/mTOR pathway (38). 3 Intermedin alleviated diabetic vascular calcification by inhibiting GLUT1 through the activation cAMP/PKA signaling pathway (42).
Pentose Phosphate Pathway (PPP)	HK2	ROS MMP hyperpolarization apoptosis suppression	1 PVT1 affects the glycolysis and phenotypic switch of VSMCs through HK2 (50). 2 Indirubin-3′-monoxime reduced HK2 expression and glycolysis in VSMCs induced by PDGF-BB (51).
	G6PD	Activation of NADPH oxidase Inflammation Apoptosis inhibition	1 Role of G6PD in tumor necrosis factor receptor-associated factor 6-induced SM22α ubiquitination and mitochondrial apoptosis inhibition through the voltage-dependent anion channel 1-Bcl-2 associated X protein pathway (54, 55). 2 Inhibiting Ca^2+^ uniporter or mitochondrial calcium uptake 1 overexpression on VSMCs from diabetic mice decreased activity of G6PD, and normalized cell proliferation (56).
Hexosamine Biosynthetic Pathway (HBP)	OGT, OGA	Upregulation of VCAM-1and RUNX2, Migration Autophagy inhibition	1 Polymerase delta interacting protein 2 deficiency enhanced OGT-mediated protein O-GlcNAcylation (63). 2 STIM1 deficiency-induced impairment of calcium homeostasis and ER stress enhances protein *O*-GlcNAcylation in VSMCs (71).
Advanced Glycation End Products (AGEs)	AGEs, RAGE	ROS Autophagy and apoptosis	1 Metformin inhibits upregulation of ALP and RUNX2 and downregulation of SM22α induced AGEs surplus (74, 80). 2 Intermedin exerts anti-calcification effects by inhibiting RAGEs via cAMP/PKA signaling pathway activation in diabetic vascular calcification (42). 3 USP10 alleviates CEL-induced vascular calcification and atherogenesis in diabetes mellitus by promoting AMPK activation (89). 4 The natural compound thonningianin A decreases expression of RUNX2, BMP2 and OPN via ATG7-dependent autophagy in HG-stimulated MASMCs (167).
Diacylglycerol-dependent PKC pathway activation	GADPH	Inducing intracellular Ca^2+^ Inflammation	1 Activation of PKC-𝜁 inducing by AGE-RAGEs activates TGF-𝛽, NF-𝜅B, and p38 MAPK, inducing VSMCs to switch their phenotype into osteoblast-like (9, 29, 79, 95–98).
Aerobic glycolysis	LDHA	Inducing fission of mitochondria mitophagy inhibition	1 Activation of κ-Opioid receptor impedes the calcification of VSMCs through decreasing lactate and PFKFB3 (116). 2 Prohibitin 2 deficiency facilitated PKM1/2 mRNA splicing and reversion from PKM1 to PKM2, and enhanced glycolysis in VSMCs (123). 3 GMRSP inhibits hnRNPA2B1-mediated alternative splicing PKM pre-mRNA, leading to reduced PKM2 production and glycolysis (124). 4 Deficiency of March2 lessened PKM2 dimer-to-tetramer conversion in VSMCs and promoted p53-driven apoptotic transcriptional response (125).

VSMCs, Vascular smooth muscle cells; GSK-3, Glycogen synthase kinase-3; TSC2/mTOR, tuberous sclerosis complex subunit 2/mammalian target of rapamycin; cAMP/PKA, activation of cyclic AMP/protein kinase A; PVT1, Plasmacytoma variant translocation 1; PDGF-BB, platelet-derived growth factor-BB; PKM, pyruvate kinase M; HK2, Hexokinase 2; GLUT 1, Glucose transporter 1; G6PD, Glucose-6-phosphate dehydrogenase; AGEs, Advanced glycation end products; RAGE, Receptor for AGEs; MMP, mitochondrial me mbrane potential; AMPK, AMP-activated protein kinase; mTOR, mammalian target of rapamycin; ROS, Reactive Oxygen Species; TCA, Tricarboxylic Acid Cycle; ATP, Adenosine Triphosphate; NADPH, Nicotinamide adenine dinucleotide phosphate; HIF-1α, Hypoxia-Inducible Factor-1α; PPAR, Peroxisome Proliferator-Activated Receptor; SIRT, Sirtuin; PI3K, Phosphatidylinositol 3-Kinase; AKT, Protein Kinase B; MAPK, Mitogen-activated protein kinase; hnRNP, heterogeneous nuclear ribonucleoprotein; March2, membrane-associated RING finger protein 2; PFKFB3, Phosphofructo-2-kinase/fructose-2,6-biphosphatase 3; GMRSP, Glucose metabolism regulatory protein; LDHA, Lactate dehydrogenase A; GADPH, Glyceraldehyde-3-Phosphate Dehydrogenase; PVTC1, Plasmacytoma variant translocation 1; STIM1, Stromal interaction molecule 1; SM22α, Smooth muscle 22 α; Atg7, Autophagy Related 7; CEL, Carboxyethyl lysine; OGT, β-N-acetylglucosaminyltransferase; OGA, β-N-acetylglucosaminidase; UPS10, Ubiquitin-Specific Protease 10; VCAM-1, vascular cell adhesion molecule-1; TGF-β, Transforming growth factor-β.

### 3.2 Upstream glycolysis overload and VSMCs osteogenic differentiation

In the upper glycolysis process, hyperglycemia disrupts glycolytic flux, causing the accumulation of intermediate metabolites such as dihydroxyacetone phosphate (DHAP), glyceraldehyde-3-phosphate (GA3P), glucose-6-phosphate (G6P), and pyruvate. 6-Phosphofructo-2-kinase/fructose-2,6-biphosphatase 3 (PFKFB3), AMP-activated protein kinase (AMPK) strictly regulates glycolytic flux. PFKFB3 - mediated glycolysis enhances the osteogenic trans-differentiation of VSMCs by modulating Forkhead box O3(FoxO3) expression and lactate generation (45). AMPK regulates glucose metabolism reprogramming and osteogenic differentiation of VSMCs by affecting pathways like PPP and HBP and increasing lactate production. High risk human carotid atherosclerotic plaques, characterized as symptomatic, vulnerable, and inflamed, were reported to exhibit enhanced glycolysis and PPP pathways and elevated amino acid utilization (45). However, little is known about the mechanisms by how VSMCs regulate this complex network. The following part mainly introduces the main metabolic links of glucose metabolism and the influence of their interactions on the calcification process of vascular smooth muscle cells.

#### 3.2.1 PPP and oxidative stress

In rat pulmonary artery smooth muscle cells (PASMCs) cultured in high glucose, the activation of the pentose phosphate pathway (PPP) affects upper glycolytic metabolites (46). Over-activation of the PPP is a key mechanism for the vascular damage related to hyperglycemia (47).

HK2, a key enzyme that phosphorylates glucose into glucose-6-phosphate, has become the predominant regulator of glycolysis in VSMCs. When high glucose levels exceed the catalytic ability of hexokinase, the surplus glucose enters the polyol pathway. These processes lead to the accumulation of reactive oxygen species (ROS), increasing oxidative stress (19, 48) (**Figure 2**). Over-expression of HK2 in human umbilical vein smooth muscle cells results in the mitochondrial membrane potential hyperpolarization and apoptosis suppression (49). Mengying Wu et al. showed that plasmacytoma variant translocation 1 (PVT1) affects the glycolysis and phenotypic switch of VSMCs through HK2 (50). Elke H. Heiss et al. found that indirubin-3′-monoxime reduced HK2 expression and glycolysis in VSMCs induced by platelet-derived growth factor (PDGF) (**Table 1**). Activation of signal transducer and activator of transcription (STAT) 3 could be identified as crucial event in upregulation of HK2 and glycolytic activity in PDGF-stimulated VSMCs (51). Xiao-Fei Gao et al. found m^6^A modification of profilin-1 interacted with the phosphorylation of ANXA2 (annexin A2) by recruiting SRC proto-oncogene, nonreceptor tyrosine kinase (Src), promoting the phosphorylation of signal transducer and activator of transcription 3 (STAT3) in VSMCs (52). Yanlin Huang et al. demonstrates that ANXA2 promotes osteogenic differentiation and inhibits cellular senescence of periodontal ligament cells (PDLCs) in high glucose conditions (53). The function of ANXA2/Src/STAT pathway on VSMCs calcification is need further study. Suppressing glycolytic enzymes such as phosphofructokinase (PFK)1 and PKM2 can redirect glycolysis towards the PPP (46). Glucose-6-phosphate dehydrogenase (G6PD) is PPP’s rate-limiting enzyme. Inhibition of G6PD in vascular cells not only prevents the over-activation of nicotinamide adenine dinucleotide phosphate (NADPH) oxidase but also reduces subsequent inflammation (47). Recent studies have highlighted the role of G6PD in tumor necrosis factor receptor-associated factor 6-induced SM22α ubiquitination and mitochondrial apoptosis inhibition through the voltage-dependent anion channel 1-Bcl-2 associated X protein (Bax) pathway (54, 55). Inhibiting Ca^2+^ uniporter or mitochondrial calcium uptake 1 overexpression on VSMCs from diabetic mice decreased activity of G6PD, and normalized cell proliferation (56; **Table 1**). Tumor protein 53 (p53) plays a role in inhibiting cancer cell proliferation and promoting apoptosis by inhibiting G6PD (57, 58). Additionally, p53 can regulate cell apoptosis by binding to VDAC1 (59, 60). However, it is still unclear whether p53 is involved in G6PD-VDAC1 mediated VSMC apoptosis.

Under high glucose conditions, the excessive activation of PPP is associated with the dysregulated regulation of HK2 and G6PD. Inhibition of 6-phosphofructokinase-1(PFK1) and PKM2 can redirect glycolysis to the PPP. Future studies could delve into the specific molecular mechanisms of ANXA2 and G6PD in glucose metabolism remodeling and calcification of VSMCs, as well as the synergistic regulatory network among key enzymes of different glucose metabolic pathways (**Figure 2**).

#### 3.2.2 HBP and protein O-GlcNAcylation

The HBP can metabolize glucose into the active O-GlcNAcylation sugar donor UDP-β-D-N-acetylglucosamine. This pathway is dynamically regulated by OGT and OGA (61) (**Figure 2**). In hyperglycemic ApoE^-/-^ mice fed a Western diet, deletion of smooth muscle-specific OGT prevents atherosclerosis development (62). F., et al. found polymerase delta interacting protein 2 (Poldip2) deficiency enhanced OGT-mediated protein O-GlcNAcylation, which inversely promoted myocardin, MRTFA (myocardin-related transcription factor A), and SRF (serum response factor) expressions (63; **Table 1**). These decreased the myocardin-dependent VSMCs marker gene expression and increased RUNX2-dependent osteogenic gene expression (64–67).

Under high glucose conditions, O-GlcNAcylation of nuclear factor-κB (NF-κB) in rat VSMCs induces upregulation of vascular cell adhesion molecule-1(VCAM-1) (68). Meanwhile, Barnes et al. demonstrated that higher O-GlcNAcylation of specificity protein 1 (sp1) in PASMCs promotes cell migration (69). Jack M Heath et al. demonstrated O-GlcNAcylation of protein kinase B (AKT) at two new sites, T430 and T479, promotes AKT phosphorylation, and then promotes RUNX2 trans-activity and VSMCs calcification (70). Besides, stromal interaction molecule 1 (STIM1) deficiency-induced impairment of calcium homeostasis and ER stress enhances protein *O*-GlcNAcylation in VSMC, which promotes VSMCs osteogenic differentiation and calcification in diabetes (71; **Table 1**). In other hand, O-GlcNAc signaling enhanced the osteogenic conversion of VSMCs through regulation of canonical Wnt/β-catenin pathway. Indeed, O-GlcNAcylation of β-catenin further increased it transcriptional activity in VSMCs (72). Besides, OGT enhances O- GlcNAcylation of kelch like ECH associated protein 1(KEAP1), leading to nuclear factor erythroid 2-related factor 2 (NRF2) degradation and subsequently inhibiting autophagy in VSMCs (73).

O-GlcNAcylation of cellular proteins such as Sp1, NF-κB, and Runx2 regulates VSMCs calcification, inflammation, migration, and the development of atherosclerosis. Future studies can further explore the specific targets and functions of O-GlcNAcylation modification, such as RUNX2.

#### 3.2.3 Increased formation of AGEs

Notably, type II diabetes patients have much higher AGEs concentrations than non-diabetics (29–31). Accumulated studies have shown AGEs surplus promote osteogenic phenotype transformation of VSMCs by increasing the levels of ALP and RUNX2 and decreasing the expression of SM22α (74–79). This effect can be inhibited by metformin (80; **Table 1**). Poetsch, F., et al. reported that AGEs increased the expression of serum and glucocorticoid-inducible kinase 1 (SGK1) and induced the osteogenic trans-differentiation of VSMCs under high glucose conditions in a NF-κB-dependent manner (81). Besides, AGEs upregulate the expression of PDK4, which inhibits the conversion of pyruvate to acetyl-CoA, ultimately reducing Kreb’s cycle flux and exacerbating calcium deposition during VSMCs calcification (7) (**Figures 2**, **3**). This effect further promotes the production of AGEs, thereby further expanding the role of AGEs in promoting VSMCs calcification.

AGEs bind to corresponding receptors, such as the receptor for AGEs (RAGE), AGE receptor 1(AGE - R1), AGE - R2, and AGE - R3, to trigger intracellular signaling cascades or exert their effects (82). RAGEs have been reported to exist in unstable plaques with microcalcifications by co-localizing with inflammatory cells and VSMCs undergoing osteochondrogenic differentiation (83). AGEs/RAGEs initiate the activation of PKC-ζ, which then activates the downstream signaling pathways mediated by p38 mitogen-activated protein kinase (p38 MAPK) and NF-κB (83, 84). (**Figure 3**). Intermedin exerts anti-calcification effects by inhibiting RAGEs via cAMP/PKA signaling pathway activation in diabetic vascular calcification (42; **Table 1**). AGEs/RAGE induce autophagy in HASMCs via the mechanistic target of rapamycin (mTOR) signaling pathway and trigger apoptosis, contributing to calcification (85). MTMR7 suppresses this effect (86). Recent study revealed that AGEs increased the activity of nuclear factor 90 (NF90), thereby promoting AGER1 degradation and ubiquitination via WD repeat domain-containing 7 and E3 ubiquitin ligase F-box’s mRNA stabilization in VSMCs induced by high glucose (87). (**Figure 3**) Therefore, AGEs receptors play important role in VSMCs calcification, death and inflammation. AGER and RAGEs may be targets to protect against VC.

Currently, carboxyethyl lysine (CEL), pentosidine, carboxymethyl-lysine (CML), and more than twenty other kinds of AGEs have been identified (88). CML was significantly increased in calcified ateries from diabetic atherosclerosis ApoE^-/-^ mice fed with high-fat diets (89). The study found that CML promoted vascular calcification through different pathways in diabetes, including p38 MAPK pathway and the nuclear factor of activated T-cells 1 (NFATc1) (90, 91). In NFATc1pathway, Protein tyrosine kinase 2 (FAK) and SIRT3 affected the nuclear translocation of NFATc1 by regulating the acetylation-phosphorylation crosstalk. Besides, CML mediates vascular calcification in diabetic plaques by impaired osteoclastic bone resorption through NFATc1-N-acetylglucosamine-1-phosphate transferase (GNPTAB) (92). Ubiquitin-Specific Protease 10 (USP10) alleviates CEL-induced vascular calcification and atherogenesis in diabetes mellitus by promoting AMPK activation (89; **Table 1**). Furthermore, CML increased the expression of PDK4 by increasing ROS (35).

#### 3.2.4 Diacylglycerol-dependent PKC pathway activation

Hyperglycemia induces accumulation of upper glycolytic intermediate glyceraldehyde-3-phosphate and increases diacylglycerol (DAG) production, which activates the PKC pathway, including PKCβ, PKCδ, and PKCα (93). G6PD can be activated by PKC to induce intracellular free Ca^2+^ to enhance the contraction of VSMCs (94). Additionally, activation of PKC-ζ inducing by AGE-RAGEs further activates transforming grow factor-β (TGF-β), NF-κB, and p38 MAPK, inducing VSMCs to switch their phenotype into osteoblast-like (9, 29, 79, 95–98) (**Figure 3**; **Table 1**).

### 3.3 Involvement of lower glycolysis overload in DR

Once pyruvate is generated, it can be converted to CO_2_ and acetyl-CoA, which enter the tricarboxylic apvt1cid cycle (TCA). Another fate of pyruvate is to be converted into lactate catalyzed by LDHA (99). The pyruvate dehydrogenase enzymatic complex (PDH) is an important link between glycolysis and the TCA (100). Reprogramming of glucose metabolism from mitochondrial oxidative phosphorylation (OXPHOS) to aerobic glycolysis has been observed during VSMCs phenotype switching (45). As regulators of mitochondrial glucose metabolism, PDKs inhibit the activity of PDH, slowing down the TCA and mediating the glucose metabolic switch of glucose metabolism from OXPHOS (100, 101). It has been made sure that PDK4 regulates VSMCs’ metabolic reprogramming towards a higher glycolysis rate, promoting lactic acid generation in the cytosol. This triggers mitochondrial dysfunction, calcium deposition and apoptosis and autophagy, inducing the osteogenic differentiation of VSMCs via direct SMAD1/5/8 phosphorylation and enhancing CBFα1 and BMP2 signaling (7, 35, 48, 102, 103).

#### 3.3.1 LDHA

LDHA is key regulatory enzyme that catalyzes the production of lactate (100). Previous studies have shown that knockout of LDHA suppresses the survival, proliferation, migration, and invasive abilities of VSMCs (104, 105). Further investigations revealed that LDHA expression is regulated by forkhead box M1(FOXM1) via binding to the LDHA promoter (106). Recent study found that YTH N6-Methyladenosine RNA Binding Protein F1 (YTHDF1) recognized vir like M6A methyltransferase associated (KIAA1429)-methylated FOXM1 mRNA and raised FOXM1 stability. This promotes the levels of glycolysis-enhancing genes (GLUT1 and LDHA) and lactate production in multiple myeloma. There is no report on whether GLUT1/LDHA regulated by stabilizing FOXM1 mRNA is involved in the calcification of smooth muscle cells.

**Figure 4:**
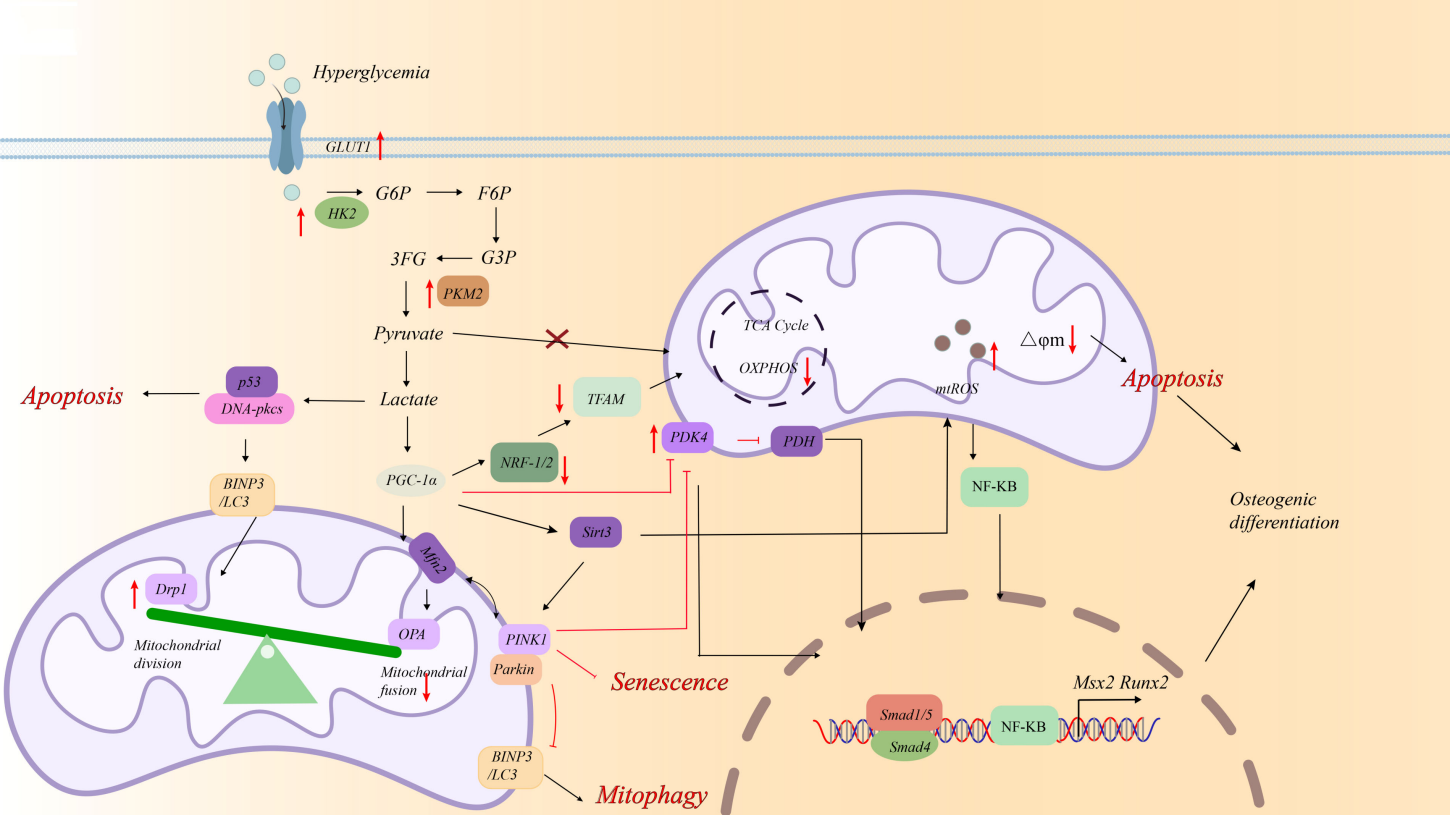
The fate and potential mechanism of VSMCs under the high glucose condition.

Upregulation of LDHA inducing surplus of lactate indue fission of mitochondria associated with dynamin-related protein 1 (Drp1) and impedes phosphatase and tensin homolog (PTEN)-induced mitophagy. PTEN-induced mitophagy mediate through putative kinase 1/parkin via the poly (ADP-ribose) polymerase 1 (PARP1)/DNA polymerase gamma (POLG)/uncoupling protein 2(UCP2) axis. Therefore, knockdown of UCP2 impedes fission of mitochondria as mediated by Drp1, while also partially restoring mitophagy via PTEN-induced putative kinase 1(PINK1)/Parkin in VSMC calcification (107) (**Figure 4**). Recent study found that PARP-1 is subjected to NEDD8 conjugation, leading to an increase in PARP-1 activity during VC. During this process, PARP-1 NEDDylation is mediated by the E3 ligase CBL proto-oncogene B (CBL-b) and is reversed by NEDD8-specific protease 1 (NEDP-1) during VC (108). Besides, lactate also accelerates VSMCs calcification through suppression of Bcl-2–19 kDa interacting protein (BNIP3)-mediated mitophagy (109). High glucose triggers lactate/G protein-coupled receptor 81(GPR81) and inhibits peroxisome proliferator-activated receptor-γ (PPAR-γ)/SIRT1 pathway, promoting vascular remodeling. HMGB2 promotes HASMC proliferation and vascular remodeling by regulating glucose metabolism through the PPAR-γ/SIRT1/PGC-1α pathway (44, 110–112). As downstream targets of the above two pathways, Peroxisome proliferator-activated receptor gamma coactivator 1-alpha (PGC-1α) overexpression can reduce ROS-mediated VSMC migration. PGC-1α/NRF2/STAT3 is involved in decreasing VC by increasing superoxide dismutase 2 (SOD2) (110–112). PPAR-γ agonists alleviated periostin-promoted VSMCs calcification and corrected abnormal glycolysis and unbalanced mitochondrial homeostasis (113). 12,13-diHOME suppressed the up-regulation of carnitine O-palmitoyltransferase 1 (CPT1A) and CPT1A-induced succinylation of HMGB1. The succinylation of HMGB1 at the K90 promoted the protein stability and induced the enrichment of HMGB1 in cytoplasm, which induced the calcification in VSMCs (114). PFKFB3 is associated with diabetic atherosclerosis and vascular remodeling through increasing lactic acid and LDHA levels (115). Activation of κ-Opioid receptor impedes the calcification of VSMCs through decreasing lactate and PFKFB3, thus becoming a possible medicinal approach and target for vascular calcification treatment (116; **Table 1**). Besides, lactate induces lactylation at the K18 site of the H3 histone protein to up-regulate chitinase 3 like 1 (CHI3L1). CHI3L1 forms a polymer complex with interleukin 13 receptor subunit alpha 2 and interleukin 13 (IL-13), activates the janus kinase 1(JAK1)/STAT3/RUNX2 signaling pathway. These findings indicate that regulating lactylation and targeting inhibition of CHI3L1 and IL-13 represent a new therapeutic strategy to reduce arterial calcification in diabetes (117).

Therefore, as an important regulatory element in lactate metabolism regulation, PGC-1α serves as a hub for cell death regulation and is involved in apoptosis, senescence, and autophagy caused by mitochondrial dysfunction (**Figure 4**).

#### 3.3.2 PKM2

The expression of PKM2 is greater in the VSMCs of atherosclerotic plaques than in those of in normal arteries (118). Tetrameric PKM2 is known to facilitate lactate production by regulating aerobic glycolysis, facilitating VSMC phenotypic switching (119). During this process, elevating crotonylation of PKM2 at K305 promotes the PKM2 dimeric form (120). Interestingly, nuclear dimeric PKM2 is implicated in the activation of the hypoxia-inducible factor-1 alpha (HIF-1α), STAT3, and β-catenin signaling pathways (121, 122). Prohibitin 2, through its C-terminus, directly interacts with heterogeneous nuclear ribonucleoprotein A1, a key modulator of PKM1/2 mRNA splicing that promotes PKM2 expression and glycolysis. Prohibitin 2 deficiency facilitated PKM1/2 mRNA splicing and reversion from PKM1 to PKM2, and enhanced glycolysis in VSMCs (123; **Table 1**). In PDGF-BB-induced synthetic VSMCs, PKM2 crotonylation (at K305) was upregulated and promotes its nuclear translocation, thereby facilitating the expression of GLUT1 and LDHA (120). Therefore, PKM2 crotonylation (at K305) may participate VSMCs calcification induced by high glucose. However, conclusive evidence on the role of PKM2 in VSMCs calcification induced by high glucose is lacking. Glucose metabolism regulatory protein (GMRSP) inhibits heterogeneous nuclear ribonucleoprotein (hnRNP) A2B1-mediated alternative splicing of pyruvate kinase M (PKM) pre-mRNA, leading to reduced PKM2 production and glycolysis. This reprogramming preserves the contractile phenotype of VSMCs and prevents their transition to a proliferative state (124; **Table 1**). Besides, membrane-associated RING finger protein 2 (March2) interacted with PKM2 to promote K33-linked polyubiquitination. Deficiency of March2 lessened PKM2 dimer-to-tetramer conversion in aortic aneurysm/dissection (AAD) and overtly exacerbated AAD-induced histone H3K18 lactylation in VSMCs by fostering glucose metabolism reprogramming, thereby promoting p53-driven apoptotic transcriptional response (**Table 1**). TEPP-46 (tetraethyl pyrophosphate), a PKM2-specific activator, pronouncedly alleviated March2 deficiency-deteriorated AAD pathology (125). Therefore, PKM2 dimer-to-tetramer conversion is one of important treatment targets.

## 4 Signaling pathways orchestrating VSMC osteogenic differentiation

The etiology of VC involves VSMCs’ osteogenic differentiation (22). The exposure of VSMCs to high glucose activates the WNT signaling pathway and BMPs via upregulation of transcription factors RUNX2 and MSX2 (126). Previous studies reveal that upregulation of MSX2 induced by high glucose increases expression of Wnt3a and Wnt7a and suppresses Dickkopf-1 (Dkk1) gene expression, which subsequently upregulates ALP and pituitary-specific positive transcription factor 1(PIT1) gene expression. These promote osteogenic trans-differentiation and calcification of VSMCs (127–130). Based on existing evidence, elevated levels of glucose participate in regulating phosphatidylinositol 3-kinase (PI3K), AMPK signaling pathway, cell death, oxidative stress and inflammation, microRNAs and extracellular vesicles, which enable chondrogenic/osteogenic phenotypic transition of VSMCs.

### 4.1 Vascular inflammation and oxidative stress

**Figure 5:**
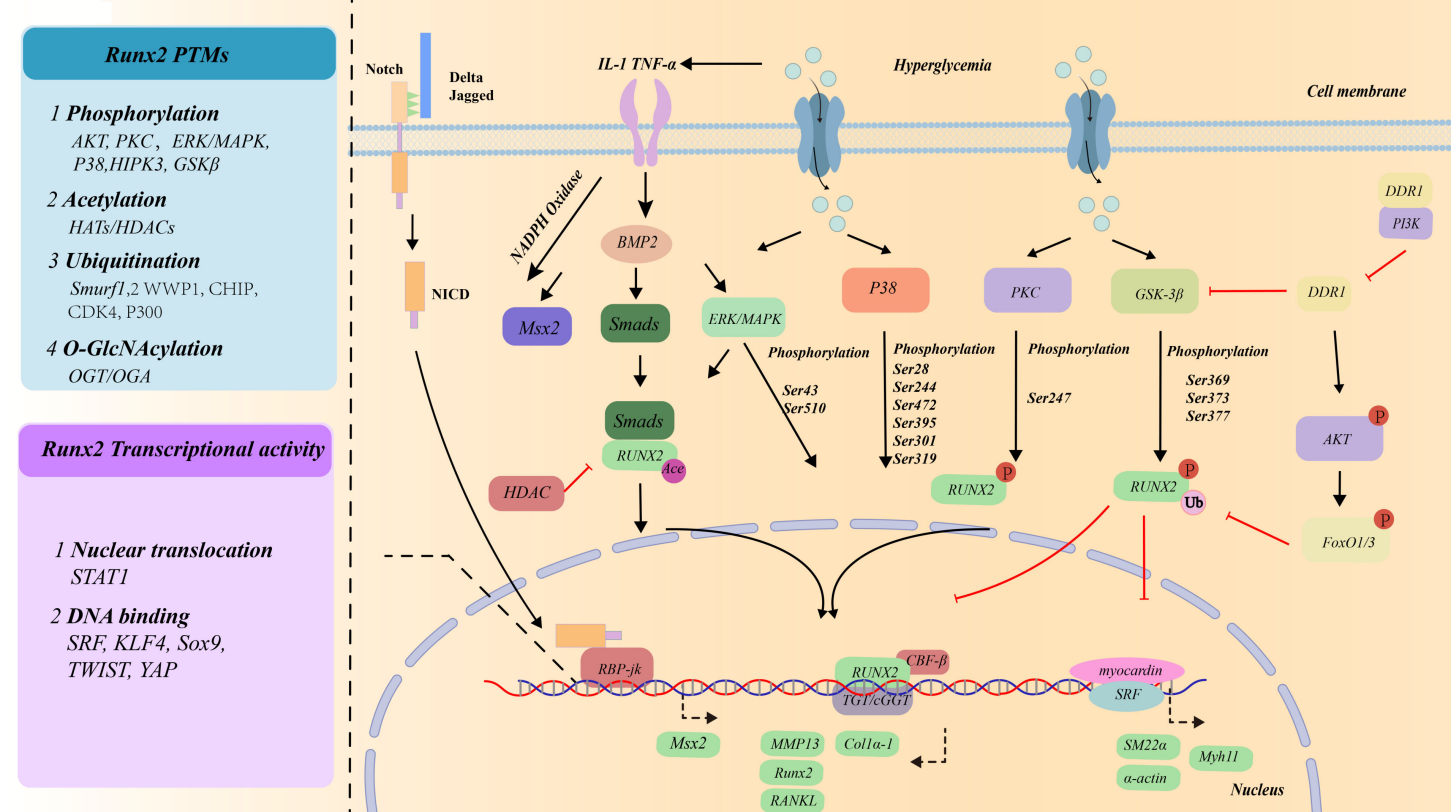
Transcriptional factors/co-factors mediating osteogenic differentiation of VSMCs under the high glucose condition.

Streptozotocin-induced diabetes increases ROS and the adventitial inflammatory response (Tumor Necrosis Factor-α(TNF-α), interleukin IL-1β, IL-6, and IL-18), playing crucial roles in osteochondral differentiation of VSMCs (131–135). This increase MSX2, BMP2, Wnt7a, and Wnt3a expressions in the aorta and accelerates calcification of the aorta (136). PGC-1α/NRF2/STAT3 is involved in decreasing VC by increasing superoxide dismutase 2 (SOD2) (110–112). In contrast, in a diabetic mouse model, vascular parathyroid hormone receptor (PTH1R) activation can also partly restrict calcification via oxidant stress reduction (32). High glucose (HG)-induced VSMC inflammation increases the level of AKT/FoxO1/3, which leads the upregulation of RUNX2 via inhibiting RUNX2 ubiquitination and subsequent degradation (65) (**Figure 5**). Oxidative stress and inflammation participate VSMCs calcification in dependent NF-kB transcriptional activation in VSMCs cultured in high glucose (68, 81, 137, 138). Accumulated studies suggest that oxidative stress and inflammatory cytokines activate monocyte chemotactic protein-1(MCP-1)/chemokine (C-C motif) receptor 2 (CCR2) and receptor of nuclear factor-κB ligand (RANKL) through RUNX2 (65, 139–143). Recent study found the leucine-rich repeat-containing G-protein-coupled receptor 4 (LGR4), a novel receptor for RANKL, also regulates VSMCs calcification (144). Mice deficient in a decoy receptor for RANKL, osteoprotegerin (OPG), develop extensive vascular calcification which is reduced by OPG treatment (145). Palmdelphin (PALMD) promoted the adjustment of glycolysis and NF-κB-mediated inflammation (137, 138). Zinc elevated TNFα-induced protein 3 (TNFAIP3) expression that was dependent on NF-κB transcriptional activation in VSMCs cultured in high glucose. This change was inhibited by zinc-sensing receptor G protein-coupled receptor 39 (GPR39) silencing (146). Empagliflozin attenuated HG-induced osteogenic differentiation and calcium deposition by increasing Bhlhe40/NLRP3 in MOVASs (147). During this process, the stability and the nuclear translocation of Bhlhe40 protein is regulated by Long noncoding RNA SNHG1 (148).

### 4.2 AMPK

AMPK is a sophisticated regulator of diabetic atherosclerosis, especially in VSMCs (149). Under high glucose condition, AMPK strictly regulates glycolytic flux via glucose uptake and mitochondrial dysfunction in VSMCs (150). For example, intermedin inhibited the expression of RAGE and GLUT1 via the cAMP/PKA signaling pathway in diabetic VC (42). Mitochondrial fission and oxidative stress enhancing VC in diabetes are blocked by the AMPK activator metformin (151–153). The glucagon-like peptide-1 receptor (GLP-1R) agonist exendin-4 promotes mitophagy by activating AMPK, inhibiting the osteogenic phenotype switching of VSMCs (5). Based on the aforementioned discussions, AMPK signaling induction is an ideal strategy in improving glucose metabolism and ameliorating diabetic complications.

### 4.3 AKT signaling pathway

HG activates of PI3K/AKT/Glycogen Synthase Kinase 3 Beta (GSK3β) (154). This process is associated with discoidin domain receptor 1(DDR1). Restoring DDR1 expression in DDR1-null VSMCs rescues Akt activation (155). In contrast, DDR1-deficient VSMCs increase GSK-3β activation and impair microtubule organization, which may account for reduced RUNX2 activity and nuclear localization (155) (**Figure 5**). In diabetic mice, AKT activation via O-GlcNAcylation enables vascular calcification induced by hyperglycemia (70, 156). AKT O-GlcNAcylation at T479 and T430 has been identified as a potential regulator of diabetes-induced calcification (70). As the AKT target, FoxO1/3 promotes RUNX2 ubiquitination and subsequent degradation, inhibits VSMCs calcification (65) (**Figure 5**). Therefore, SMC-specific tensin homologue (PTEN) (a primary negative AKT regulator)/FOXO promotes RUNX2 upregulation and VSMCs calcification (157). Sal B exhibits substantial anti-inflammatory effects by modulating the miR-486a-5p/FOXO1 axis under HG conditions in VSMCs (158). Besides, GLP-1R mediates VSMCs calcification in diabetes through inhibiting ERK1/2 and PI3K/Akt signaling pathways (159).

### 4.4 Cell fate

High glucose triggers autophagy, apoptosis, defect mitophagy and senescence. This finding underscores the pivotal role of metabolic disturbances in vascular pathology (160). High glucose triggers autophagy in VSMCs through two distinct signaling pathways: HIF-1α/PDK4 axis and the cyclic guanosine monophosphate (cGMP)-protein kinase G (PKG) pathway (161, 162) (**Figure 4**). These partly contribute to AGEs accumulation (163, 164). Notably, Hu-Qiang He et al. confirmed that impaired autophagy effectively inhibited AGE-induced calcification in HASMCs (85). Xu, Z.J., et al. showed autophagic dysregulation is mechanistically also governed by the Notch receptor 3 (NOTCH3)/RAN binding protein 1(RANBP1) axis in high glucose-stimulated VSMCs (165). Dickkopf1 (Dkk1) slowed vascular calcification by promoting the degradation of PLD1 via the regulating autophagosome formation and maturation (166). The natural compound thonningianin A (TA) decreases expression of RUNX2, BMP2 and OPN via autophagy related 7 (ATG7)-dependent autophagy in HG-stimulated MASMCs (167; **Table 1**). AGEs also facilitate apoptosis to release more matrix vesicles and establish a calcium–phosphorus deposition microenvironment (168). The enhanced expression of HK2 in HASMCs results in the hyperpolarization of mitochondrial membrane potential and the suppression of apoptosis (49). PKM2 dimer-to-tetramer conversion foster glucose metabolism reprogramming and promote p53-driven apoptotic transcriptional response (125).

High glucose promotes surplus of lactate. Accumulated studies confirmed that lactate paly a core role in regulated cell fate in diabetes. In one hand, lactate accelerates Drp1-regulated fission of the mitochondria. On the other hand, lactate inhibits mitophagy via BNIP3 and PTEN-PINK1/Parkin. All promote the osteoblastic phenotype transition of VSMCs and calcium deposition (151). PARP1 knockdown inhibited Drp1-mediated mitochondrial fission and partially restored PINK1/Parkin-mediated mitophagy. Further study found lactate promote PARP1 expression and nuclear transfer and then activate POLG/UCP2 pathway, inhibiting mitochondrial DNA synthesis (107). Metformin reduces the advancement of diabetes-induced atherosclerosis by blocking Drp1-mediated mitochondrial fission (152) (**Figure 4**). The glucagon-like peptide-1 receptor (GLP-1R) agonist exendin 4 (EX4) inhibited osteogenic differentiation of HG/β-GP-induced VSMCs through promoting mitophagy by activating the AMPK signaling pathway (5).

VSMCs isolated from diabetic patients show elevated DNA damage and senescence. PGC-1α plays a causative role in the pathogenesis of senescence (169). Hyperglycaemic conditions induced DNA damage and enhanced senescence in VSMCs *in vitro*. CML caused these changes via stimulator of interferon response cyclic guanosine monophosphate - adenosine monophosphate (cGAMP) interactor 1 activation (170). DNA damage-induced calcification is accelerated within a diabetic environment and can be attenuated *in vitro* by SIRT1/ATM activation (171). DNA damage and senescence promote vascular calcification through SIRT1/PGC-1α pathway in VSMCs (44). PGC-1α and O- GlcNAcylation of KEAP1 lead to NRF2 degradation, thereby inhibiting the negative regulatory effect of NRF2 on the stability of STING mRNA and ultimately promoting STING expression (110–112, 172). Besides, Hyperglycemia stimulates vascular endothelial cells to upregulate cyclin-dependent kinase inhibitor 1A (p21) and p53, thereby exacerbating VSMC senescence and calcification (173).

### 4.5 miRNAs that regulates VSMC osteogenic differentiation

MicroRNAs (miRNAs) are small non-coding RNAs approximately 22 nucleotides in length. These molecules bind to complementary seed sequences located within the 3’ untranslated region (3’UTR) of target messenger RNAs (mRNAs). MicroRNAs have emerged as crucial post-transcriptional regulators in the process of VSMCs re-differentiation from contractile to osteoblast-like phenotype in diabetics (174).

miRNAs effectively silence gene expression, such as RUNX2, MSX2. Overexpression of miR-34a activates NF-κB, subsequently increasing the expression of RUNX2 and osteocalcin (175–177). Meanwhile, microRNA-126-3p impedes the osteogenic trans-differentiation of VSMCs by post-transcriptionally interfering with BMP2 gene expression (178). High glucose levels were shown to induce the upregulation of miR-32-5p by activating CCAAT/enhancer binding protein beta (CEBPB). Overexpression of GATA6 antagonized the effects of miR-32-5p on vascular calcification (179). By modulating the translation efficiency or stability of their target mRNAs, miRNAs are involved in key functions including cell growth, proliferation, differentiation, metabolic regulation, immune responses, and the regulation of cell death pathways (180). In high glucose conditions, the expression of miR142-3p (181), miR21-5p (182), and miR19a (183) are promoted. In contrast, miR24 (184) and miR9 (185) expression is inhibited. These miRNAs participate in regulating proliferation and migration of VSMCs. For example, miR-24 overexpression can mitigate proliferation of VSMCs in diabetic rats through the Wnt4/Dishevelled-1(Dvl-1)/β-catenin signaling pathway (186). Decreasing miR125a expression promotes VSMCs migration and proliferation by upregulating 3-hydroxy-3-methylglutaryl coenzyme A reductase (187). Additionally, miR210 inhibits the carbohydrate-responsive element-binding protein (ChREBP)/HIF-1α signaling pathway, regulating glycolysis and apoptosis (188). The binding of RNA-ES3 to Bhlhe40 suppresses the expression of miR-6776-5p, miR-95-5p, miR-4747-5p, and miR-3620-5p, leading to VSMCs senescence and calcification (189). In contrast, MiR-15a/15b/16 specifically target the 3’UTR of nuclear factor of activated T cells (**3**NFATc3) mRNA, and downregulate OCN expression. Therefore, MiR-15a/15b/16 inhibits human VSMCs osteogenic trans-differentiation (162).

Matrix vesicles and circulating miRNAs also play an important role in VSMCs calcification. A deficiency in Hsa_circRNA_0008028 exacerbates high glucose-induced calcification, autophagy, and proliferation in VSMCs by upregulating miR-182-5p. During this process, miR-182-5p targets tribbles pseudokinase 3 (TRIB3). TRIB3 plays a critical role in the induction and maintenance of contractile phenotype in VSMCs (180). Similarly, extracellular vesicles (EVs) carrying circ_0008362 elevated in diabetic patients. It increases the expression of RUNX2 through miR-1251-5p in VSMCs (190). Additionally, macrophage-derived miR-32 inhibits autophagy in type 2 diabetic mice’s arterial calcification (162). And MAEC-derived exosomal circHIPK3 increases high glucose-induced VSMCs proliferation via the miR-106a-5p/Foxo1/vascular cell adhesion molecule 1(VCAM1) pathway (190).

### 4.6 EVs

Mineral homeostasis disruption under high glucose conditions is closely associated with the dysregulation of EVs, including exosomes and matrix vesicles (MVs) (191). Emerging evidence highlights the critical role of EVs in mediating vascular calcification exacerbated by hyperglycemia (192). Therefore, EVs may be potential therapeutic targets in diabetic vascular complications.

#### 4.6.1 EVs of VSMCs: high glucose-induced pro-calcific transformation

Extracellular vesicles coming from VSMCs contain calcification-promoting protein tissue-nonspecific alkaline phosphatase (TNAP) (193) and sortilin (194).The sortilin 1 (SORT1) and neutral sphingomyelinase 2 (nSMase2, also called sphingomyelin phosphodiesterase 3; SMPD3) are crucial enzymes in the generation of EVs and cargo sorting (195). Treatment with anti-sortilin antibodies may considerably lessen EV-induced VC (196). Inhibiting nSMase2 pharmacologically decreases VSMCs EVs secretion and VSMC-driven calcification (197, 198). High quantities of Nϵ-carboxymethyl-lysine increased release of EVs coming from VSMCs and recruitment of sortilin to EVs (196). Additionally, galectin-3 overexpression in macrophages, triggered by high glucose, facilitates the migration of VSMC-derived EVs to the intima, further exacerbating diabetic vascular intimal calcification (199).

#### 4.6.2 EVs of ECs: bidirectional regulation under high glucose stimulation

Through EVs, endothelial cells (ECs) can communicate with VSMCs (200). High glucose-exposed human umbilical vein endothelial cells (HUVECs) secrete EVs that act as key mediators in VSMC calcification. These EVs deliver Notch3 to VSMCs via the mTOR signaling pathway, promoting VSMCs phenotypic transition towards an osteoblast-like state (201). HG-HUVECs-EVs also contain versican (VCAN), which induce mitochondrial dysfunction, oxidative stress and senescence, which accelerates VSMCs calcification (173). Moreover, high glucose induces the production of EC-derived EVs containing malondialdehyde (MDA), LDH, CircRNA-0077930, and circ_0008362, which promote VSMCs calcification (173, 190, 201–203). Notably, contrary to earlier research suggesting that AGEs were detrimental to vascular cells, Guo et al. demonstrated that AGEs might actually prevent diabetic media calcification by inducing HUVECs to release small EVs carrying abundant miR-126-5p. This miRNA targets bone morphogenetic protein receptor type 1 (BMPR1) and blocks the SMAD1/5/9 signaling pathway (203).

#### 4.6.3 EVs of macrophages: molecular link between pro-inflammation and pro-calcification in high glucose environment

**Figure 6:**
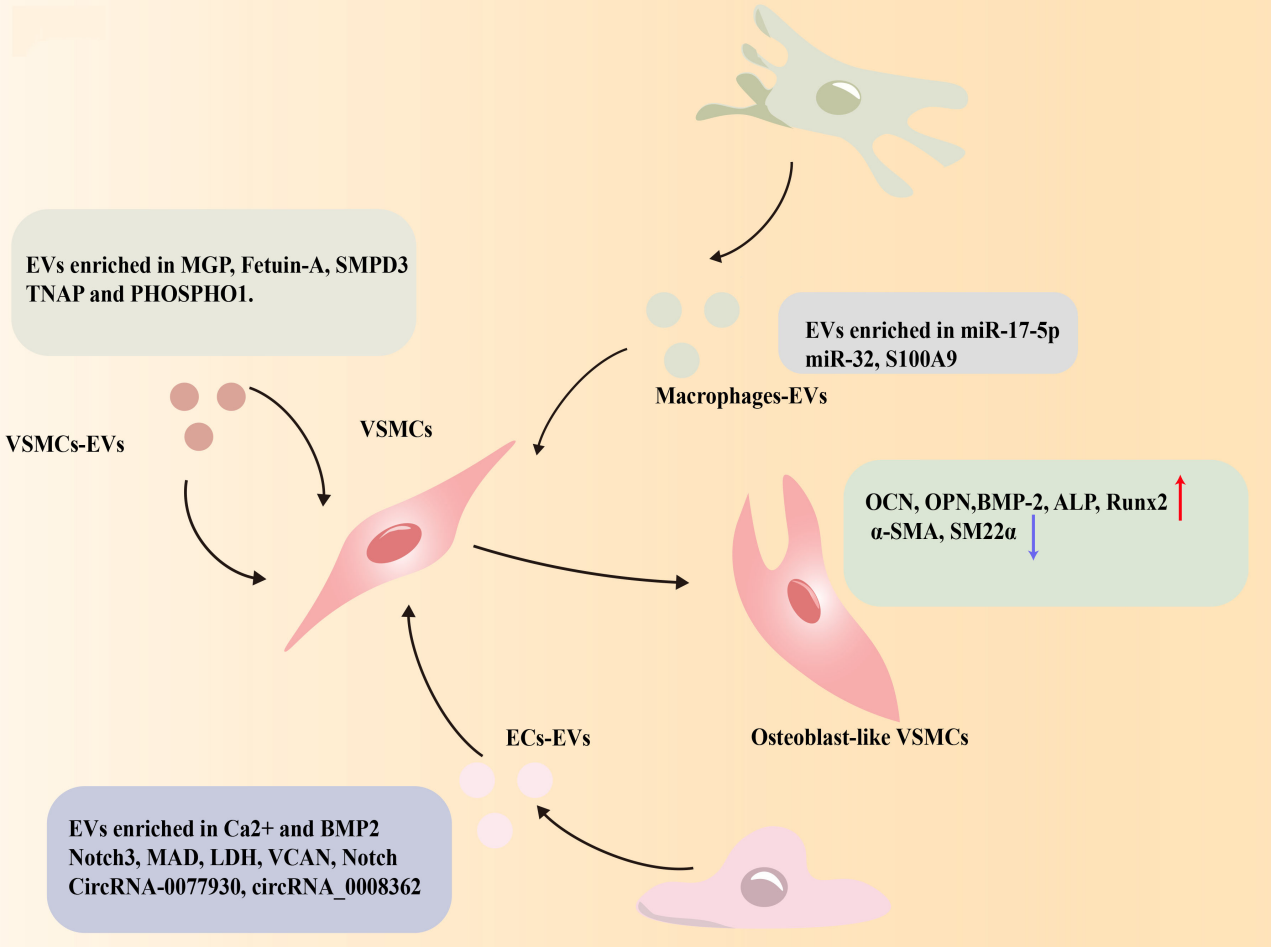
Potential mechanism of EVs regulating osteogenic differentiation of VSMCs under the high glucose condition.

In the high glucose-induced inflammatory microenvironment, macrophage-derived EVs play a pivotal role in VSMCs osteogenic differentiation. MiR-17-5p, macrophage S100A9 and miR-32 are derived from EVs of macrophage. MiR-17-5p attenuates VSMCs osteogenesis through suppressing TGF-β signaling (204). Macrophage S100A9 controls diabetic vascular calcification via NRF-2 and NF-κB (205) (**Figure 6**). MiR-32 accelerates vascular calcification in type 2 diabetes by inhibiting VSMCs autophagy (162).

## 5 Conclusions and future perspectives

Vascular calcification has emerged as a vital mortality or morbidity indicator in the cardiovascular system, particularly in diabetes. Osteogenic differentiation of VSMCs is considered a significant mechanism of diabetic macrovascular disease and the initiation mechanism of vascular calcification (65, 206–208). HG activates multiple signaling pathways, like Ca^2+^ signaling, Wnt/β-catenin, BMP/Smad, and Notch through AMPK, PI3K-AKT, oxidative stress and inflammation, microRNA, and EVs pathways (209–211). These activations contribute to glucose metabolic reprogramming such as (a) intracellular PKC overactivity; (b) increased polyol pathway flux; (c) increased the hexosamine pathway flux; and (d) generation of AGEs and other glycated compounds derived from both glucose oxidation in arterial VSMCs (212). These changes promote osteogenic transformation of VSMCs and the development of VC.

RUNX2 is the core of the regulatory network for VSMCs calcification. During glucose metabolism reprogramming, PPP, HBP, and AGEs/AGER activate ERK1/2, MAPK, AKT, NF-κB and BMP2 in VSMCs. These pathways mainly participate in RUNX2 expression and transcriptional activity through regulate RUNX2 protein structure, and the multilateral genetic, epigenetic and posttranslational modifications (PTMs) regulatory mechanisms control RUNX2 expression (213) (in **Figure 2**). Further investigations are warranted to address these unanswered questions, which should provide new breakthroughs in the understanding of RUNX2-dependent transcriptional reprogramming of vascular cells and their contributions to the development of arteriosclerosis. For example, it is unknown whether RUNX2 is upregulated in all VSMCs or exclusively in selective VSMCs subpopulations. Of particular interest, RUNX2-regulated expression of RANKL by VSMCs functions as a chemoattractant that induces macrophage/monocyte migration towards VSMCs; and further induces the differentiation of the macrophage/monocyte into bone-resorbing osteoclast-like cells.

Under the high glucose condition, the expression of GLUT, HK2, G6PD, PKM2 and LDHA increase, which enhances glycolytic activity and decreases OXPHOS in VSMCs. During VSMCs calcification, upregulation of PKM2 promote the expression of GLUT1 and LDHA (120). PDK4 inhibit the activity of PDH, slowing down the TCA and towards a higher glycolysis rate. Besides, GLUT1/LDHA may regulate aerobic glycolysis. These explain the preference of VSMCs for the less ATP-efficient metabolic mode of glycolysis. Future efforts will likely focus on identifying key regulatory points and interactions between mechanisms linked to VSMCs transformation that can be targeted to reduce calcification, and, thereby, improve vascular compliance and reduce cardiovascular risk.

There are still many difficult problems to be solved in future research. Specifically, the representation of cultured cells in *in vivo* studies is limited because of the lack of a suitable microenvironment, such as cell–cell interactions, extracellular elastin fibers, hemodynamic factors, and cytokines (214–216). In addition, animal models cannot successfully recapitulate the physiology and pathology in humans. Therefore, the trend of VSMCs transformation in diabetic patients and metabolic reprogramming remains to be further explored and has recently attracted attention.
